# Utilisation of Ambient Laser Desorption Ionisation Mass Spectrometry (ALDI-MS) Improves Lipid-Based Microbial Species Level Identification

**DOI:** 10.1038/s41598-019-39815-w

**Published:** 2019-02-28

**Authors:** Simon J. S. Cameron, Zsolt Bodai, Burak Temelkuran, Alvaro Perdones-Montero, Frances Bolt, Adam Burke, Kate Alexander-Hardiman, Michel Salzet, Isabelle Fournier, Monica Rebec, Zoltán Takáts

**Affiliations:** 1 Division of Computational and Systems Medicine, Department of Surgery and Cancer, Imperial College London, South Kensington Campus, London, UK; 2 The Hamlyn Centre, Department of Computing, Faculty of Engineering, Imperial College London, South Kensington Campus, London, UK; 3 Laboratoire Proteomique, Reponse Inflammatoire et Spectrometrie de Mass (PRISM), Universite de Lille, Lille, France; 4 Department of Microbiology, Imperial College Healthcare NHS Trust, Charing Cross Hospital, London, UK

## Abstract

The accurate and timely identification of the causative organism of infection is important in ensuring the optimum treatment regimen is prescribed for a patient. Rapid evaporative ionisation mass spectrometry (REIMS), using electrical diathermy for the thermal disruption of a sample, has been shown to provide fast and accurate identification of microorganisms directly from culture. However, this method requires contact to be made between the REIMS probe and microbial biomass; resulting in the necessity to clean or replace the probes between analyses. Here, optimisation and utilisation of ambient laser desorption ionisation (ALDI) for improved speciation accuracy and analytical throughput is shown. Optimisation was completed on 15 isolates of *Escherichia coli*, showing 5 W in pulsatile mode produced the highest signal-to-noise ratio. These parameters were used in the analysis of 150 clinical isolates from ten microbial species, resulting in a speciation accuracy of 99.4% - higher than all previously reported REIMS modalities. Comparison of spectral data showed high levels of similarity between previously published electrical diathermy REIMS data. ALDI does not require contact to be made with the sample during analysis, meaning analytical throughput can be substantially improved, and further, increases the range of sample types which can be analysed in potential direct-from-sample pathogen detection.

## Introduction

The rapid and accurate identification of the causative organism of an infection is fundamental in ensuring that a patient receives the most appropriate and effective treatment, in the shortest possible timeframe. Traditional methods of identification in clinical microbiology were reliant upon morphological and biochemical tests, which were costly, time-consuming, and often required multiple days to complete^1,2^. In recent years, the introduction of various matrix assisted laser desorption ionisation spectrometry (MALDI) platforms has substantially reduced the time to identification over traditional methods^3–6^. However, commercially available MALDI platforms, such as the MALDI Biotyper (Bruker) and VITEK MS (bioMerieux), require user input to transfer microbial biomass to an analysis plate and deposit a matrix to enable the ionisation of proteins. Furthermore, some microorganisms, such as yeasts and Gram-positive bacteria require lengthy protein extractions for reliable identifications^3,7,8^. Ambient ionisation mass spectrometry (MS) offers alternatives to currently used MS platforms to improve analytical throughput. For example, desorption electrospray ionisation mass spectrometry (DESI-MS)^9^, liquid extraction surface analysis mass spectrometry (LESA-MS)^10^, and swab-spray mass spectrometry^11^ have demonstrated the ability to differentiate bacteria directly from culture with no preparative steps, albeit using a small number of species.

The ambient ionisation method rapid evaporative ionisation mass spectrometry (REIMS) offers an alternative to currently used approaches as it allows the direct-from-culture analysis of microorganisms; without the requirement for sample preparation nor extraction. REIMS is a rapid ionisation method where thermal disruption of a biomass produces an aerosol containing gas-phase ions of metabolites and complex lipids. As with other ambient ionisation MS methods, REIMS-based microbial identification utilises the complex lipid region signal, typically the 600 to 1000 *m/z* mass range. Interestingly, the analysis of lipids was one of the first methods for microbial identification using mass spectrometry^12^ and has been utilised by a variety of different approaches including gas chromatography mass spectrometry^13–15^, fatty acid methyl ester analysis^16,17^, and fast atom bombardment mass spectrometry^18^.

**Figure 1:**
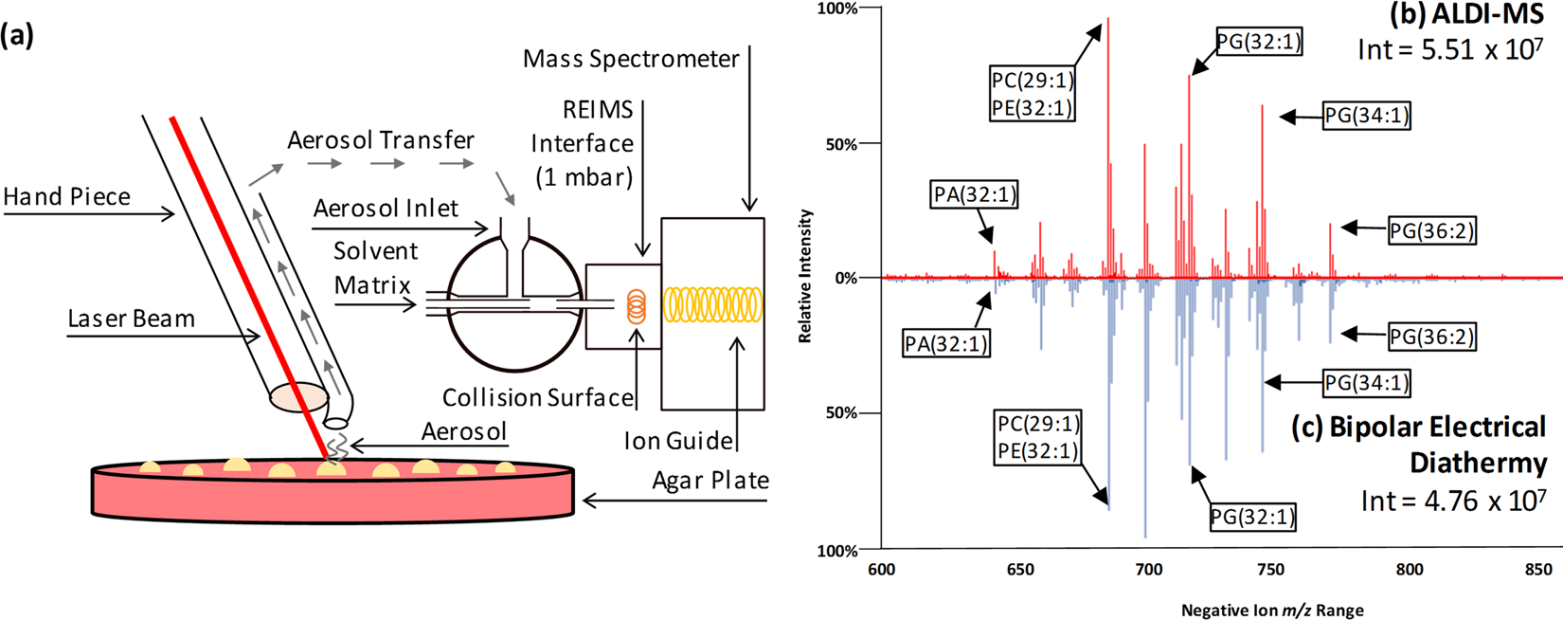
Experimental Set-Up and Representative Mass Spectra. (**a**) Experimental set-up of handheld CO_2_ ALDI-MS of microbial biomass from culture, and mass spectrometry analysis. Figure not drawn to scale. Representative mass spectra of the complex lipid region (600 to 850 *m/z*) of an *Escherichia coli* isolate analysed using (**b**) ALDI-MS and (**c**) handheld bipolar electrical diathermy. Putative identifications of complex lipids completed through accurate mass interrogation of the LIPID MAPS database with the highest scoring match (based on Delta score) used as putative identification.

For chemical analysis using REIMS, the generated aerosol is directly introduced to a mass spectrometer, after mixing with a solvent matrix which has previously been shown to improve signal intensity^19^, via an atmospheric interface containing a heated collision surface (Fig. 1). The heated collision surface acts to break apart ion clusters so that individual species enter the mass spectrometer for analysis. The initial utilisation of REIMS analysis employed a radiofrequency alternating electrical current to rapidly heat a biomass, producing an analyte-containing aerosol^20,21^. For microbiology applications, REIMS has been utilised previously using both a handheld bipolar probe^22,23^ and an automated, high-throughput robotic platform employing a monopolar probe^19,24,25^. The use of an electrical current however, necessitates contact to be made between the probe and biomass; resulting in the requirement for either cleaning or changing of the probe between analyses. The combination of laser ablation and mass spectrometry was first reported over 40 years ago, such as in the form of the laser microprobe mass analyser (LAMMA)^26^. It has previously been shown that electrical diathermy and radiative infrared laser heating provide identical mass spectra^20^. The utilisation of laser desorption will increase analytical throughput as no contact is required between the sample and the electrical probe, removing the necessity for cleaning and/or changing of probes between sample analyses.

Here, we report on the first use of ALDI mass spectrometry analyse 150 microbial isolates, including the assessment of various operational parameters of the instrument. This resulted in improved sample throughput and taxonomic classification accuracy; particularly with regards to microbial species producing low biomass during growth.

## Results and Discussion

The use of REIMS as a potential tool in diagnostic microbiology has, to date, utilised electrical diathermy to thermally disrupt biomass, creating a vapour containing analytes in gas-phase. Ultraviolet and infrared lasers have previously been used to analyse biological tissues^27^, but have not been used in the analysis of microorganisms. To determine the efficacy of using atmospheric laser desorption ionisation as a mechanism for thermal disruption of microbial biomass directly from an agar plate, this study analysed 150 isolates from 10 microbial species. The microbial species contained both Gram-negative and Gram-positive bacteria, and one fungal species. These represent common disease-causing pathogens, such as *Escherichia coli* and *Staphylococcus aureus*, and species which are frequently isolated in diagnostic laboratories, such as *Lactobacillus jensenii*, but which rarely cause disease.

### Optimisation of ALDI-MS Analytical Set-Up

**Figure 2:**
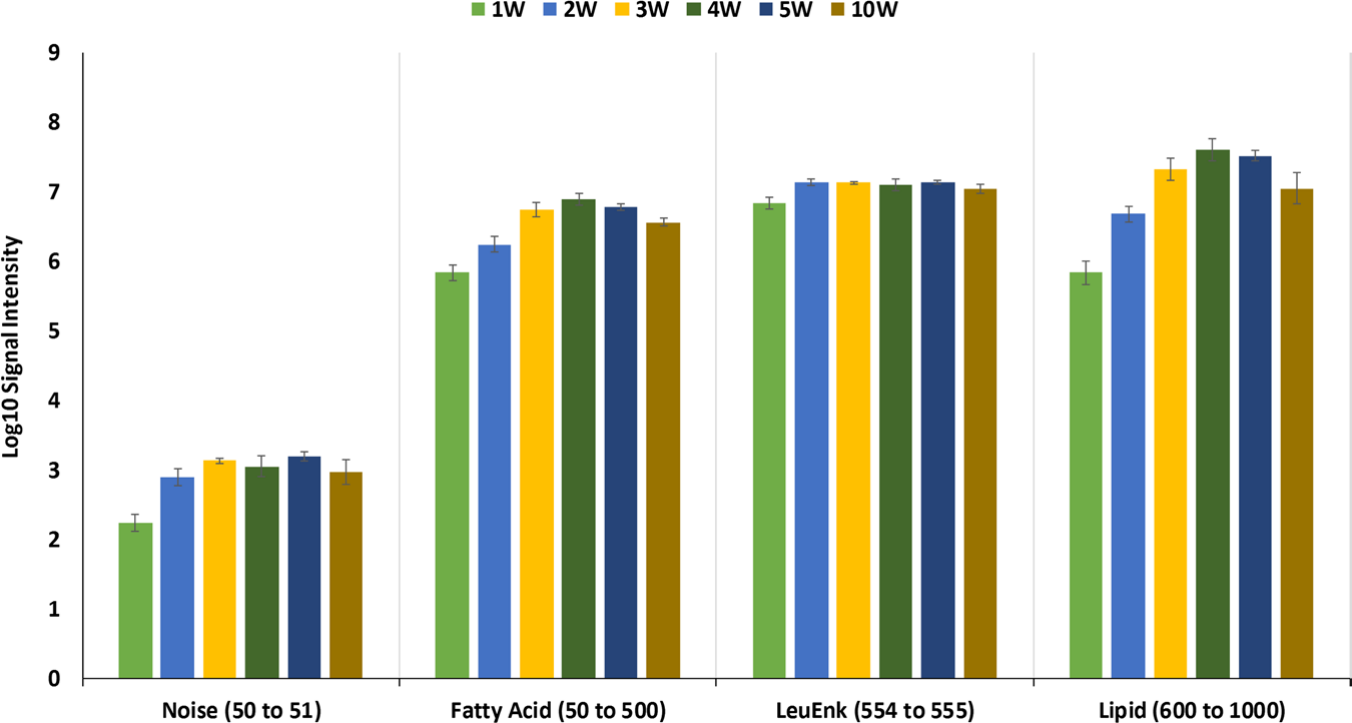
Signal Intensity for ALDI-MS Heating Power Used in Optimisation. Average signal intensities for each of the six heating powers used during initial optimisation are given as a mean for all 15 isolates of *Escherichia coli*. Error bars indicate one standard deviation around the mean.

**Figure 3:**
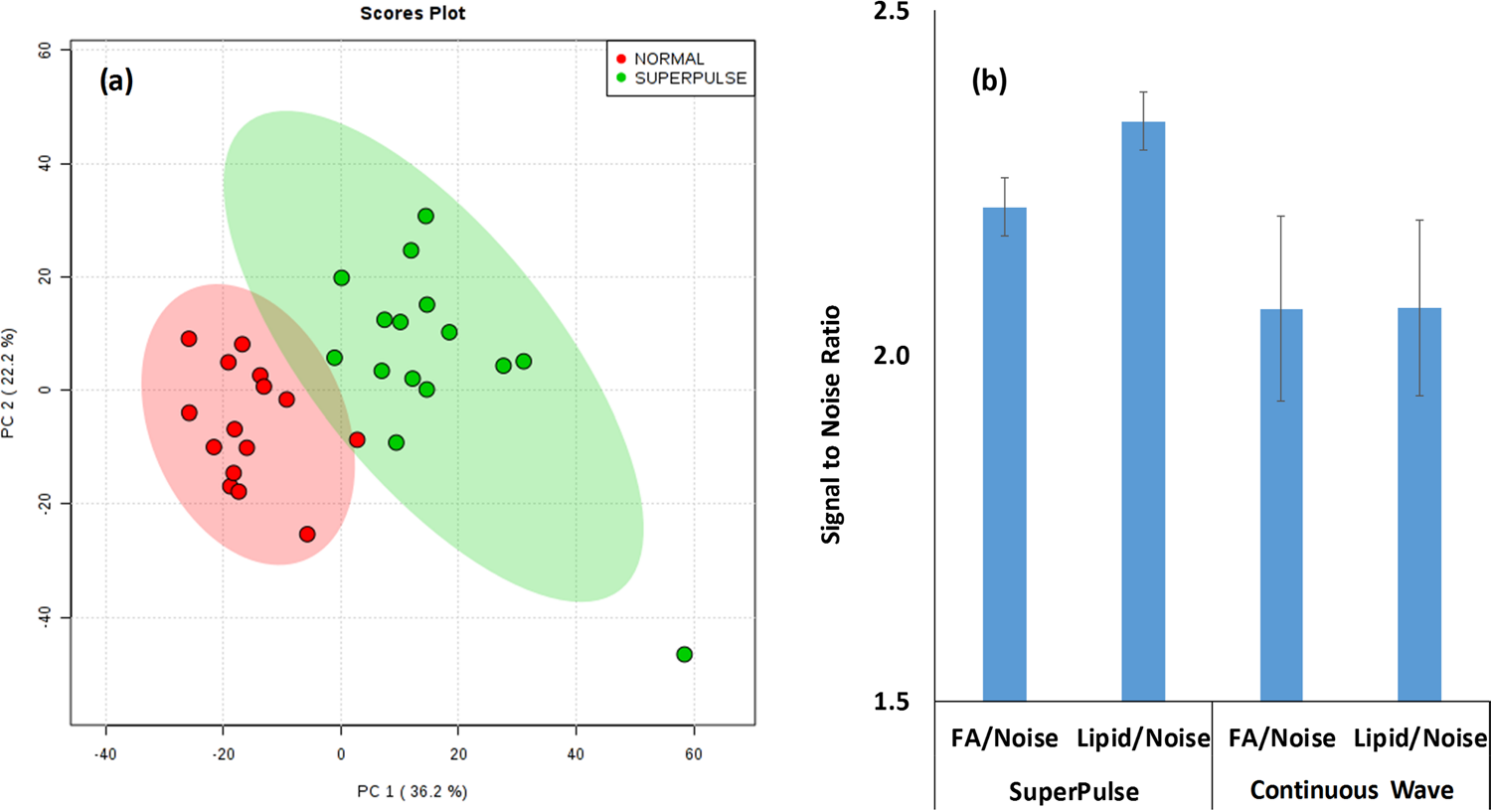
Comparison between Continuous Wave and SuperPulse Pulsatile Operational Modes. (**a**) Principal component analysis of the mass spectral data generated using both continuous pulsatile (red) and superpulse pulsatile (green) modes of the FELS-25A CO_2_ laser on 15 isolates of *E. coli* shows clear and significant separation between the mass spectra (50 to 2500 *m/z* range) acquired using the two different modalities. (**b**) Mean signal-to-noise ratios of two mass spectral regions (FA = 50 to 500 *m/z* range of fatty acids and low molecular weight metabolites/Lipid = 600 to 1000 *m/z* range of phospholipids/Noise = 50 to 51 *m/z* range). Error bars indicate one standard deviation around the mean.

Initial optimisation of the laser operational parameters, including power and pulse modes was conducted on 15 *Escherichia coli* isolates using the experimental set-up shown in Fig. 1. This included the heating power (1 W to 5 W in 1 W increments and 10 W) and continuous wave operation (which emits a continuous laser beam with a controlled output) against pulsatile (referred to as SuperPulse) operation (which sends short, high peak power pulses, with breaks in between pulses, but which give the same total energy per second) mode. Signal intensity values were calculated for four spectral regions including noise (50 to 51 *m/z*), fatty acid and low molecular weight metabolites (50 to 500 *m/z*), leucine encephalin used as an external lock mass compound (554 to 555 *m/z*), and phospholipids (600 to 1000 *m/z*), shown in Fig. 2. This showed that a plateau in signal intensity was achieved at 4 W to 5 W heating power, and thus a heating power of 5 W was used for subsequent analyses. Additionally, the effect of pulsatile mode was assessed through analysis of the same 15 *E. coli* isolates. Figure 3a shows clear and significant differences between mass spectra acquired with superpulse mode on or off, with Fig. 3b showing that the superpulse heating mode acquired mass spectra with a significantly higher signal to noise ratio for both the fatty acid and phospholipid regions. Thus, superpulse heating mode was used in the subsequent analysis of all 150 isolates. Figure 1b shows representative mass spectra within the 50 to 1000 *m/z* range, in negative ion detection mode, of an *E. coli* isolate acquired after laser operation optimisation, in comparison to mass spectra acquired using electrical diathermy REIMS as previously described^19^. From this, many of the same spectral features are visible between both aerosolization modalities, particularly within the phospholipid region of 600 to 800 *m/z*; suggesting that both modalities follow similar mechanisms of ion formation, as previously described^20^. This is further supported by putative identifications, based on accurate mass measurements, from the LIPID MAPS database^28^, of phospholipids within the mass spectra, Fig. 1b,c, showing the same features are present. These include common microbial cell membrane lipids including phosphatidic acids, phosphatidylethanolamine, and phosphatidylglycerols.

### Microbial Speciation using Optimised ALDI-MS Analytical Set-Up

**Figure 4:**
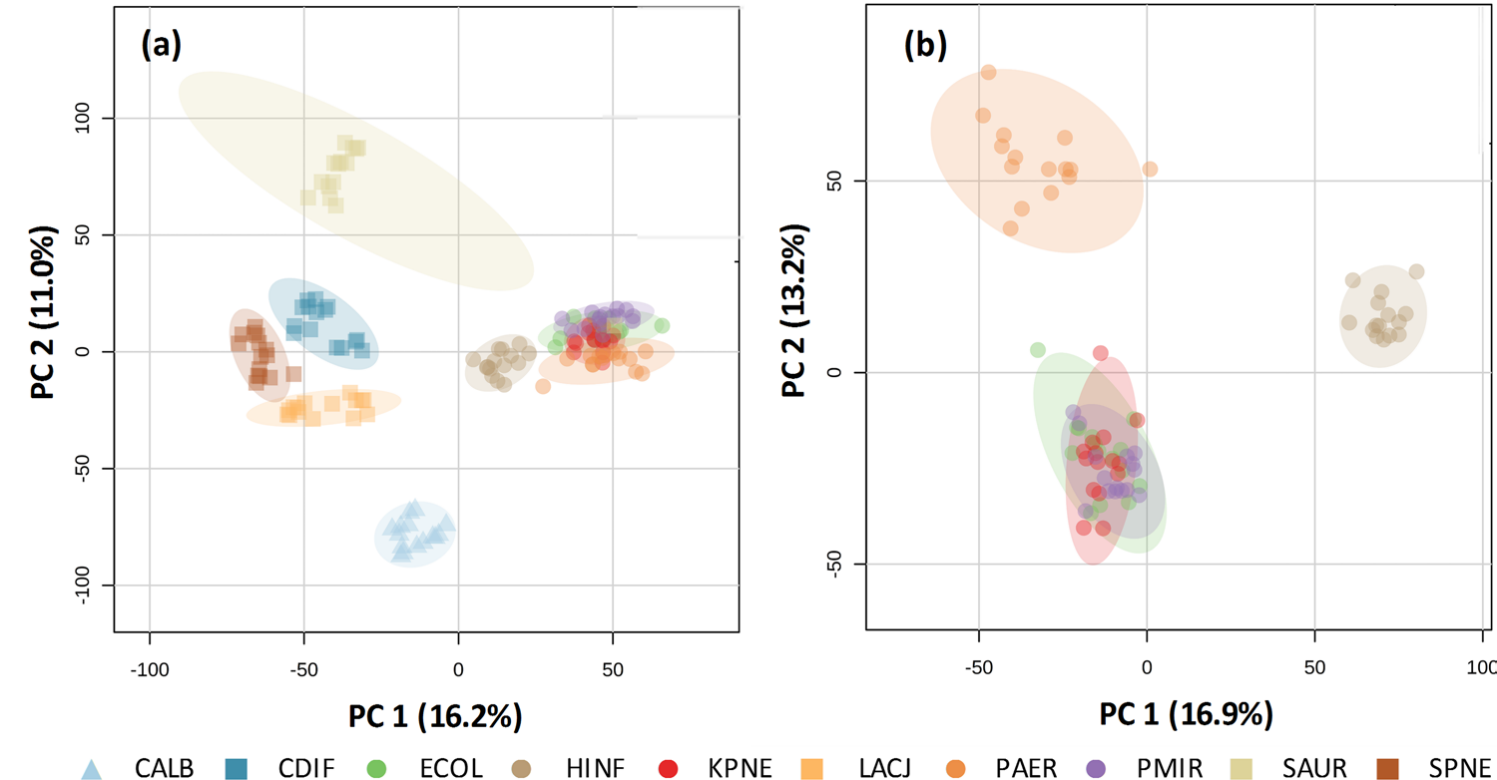
Principal Component Analysis of Isolates Analysed using ALDI-MS. Mass spectral data of 150 isolates is shown through unsupervised principal component analysis modelling of the 600 to 1000 *m/z* mass range for (**a**) all microbial species analysed in study, and (**b**) five Gram-negative species analysed in study. Shaded regions indicate 95% confidence intervals of statistical separation. Triangle symbol indicates yeast, square indicates Gram-positive morphology, and circle indicates Gram-negative morphology.

**Figure 5:**
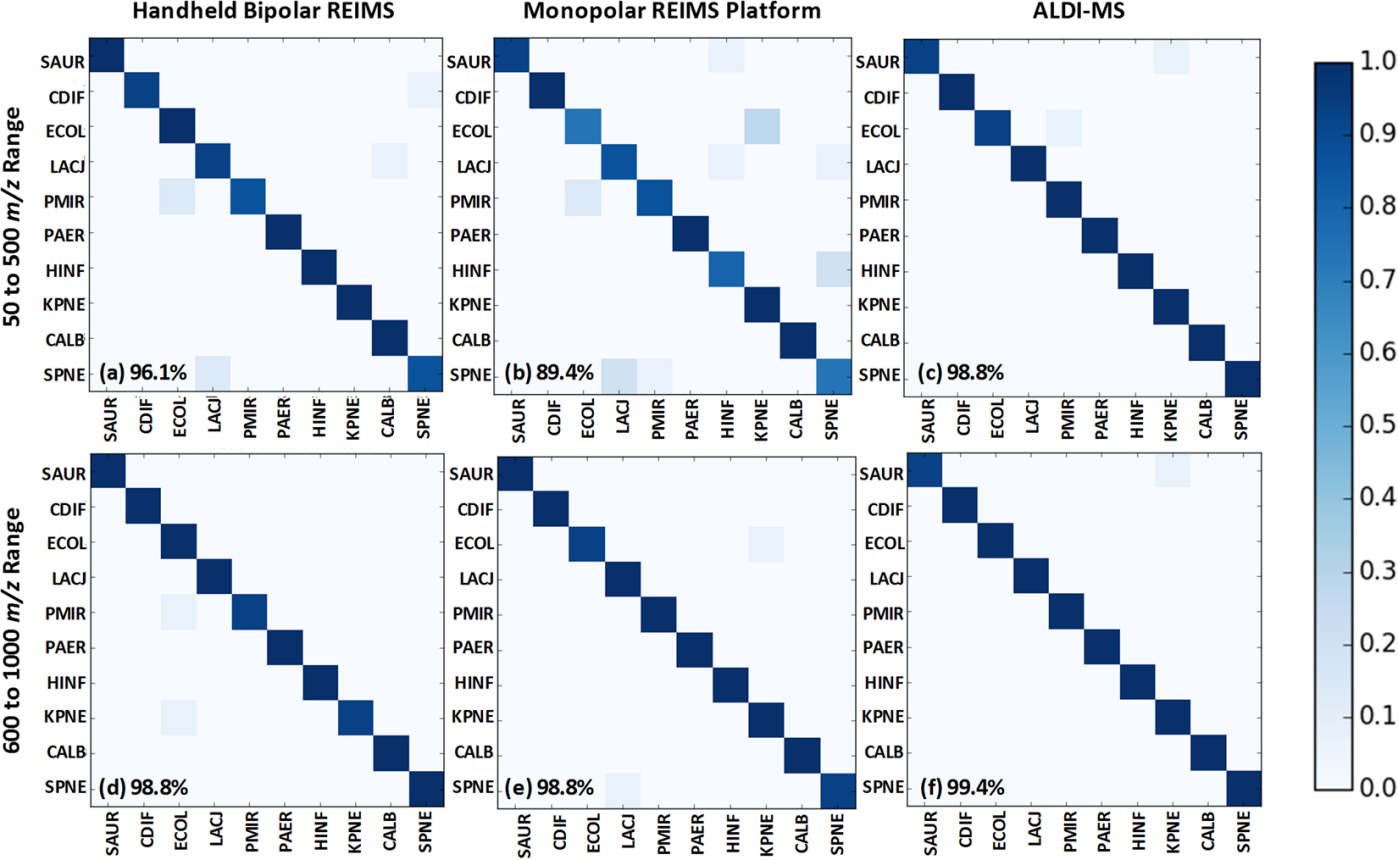
Random Forest Classification Models using Three Ambient Modalities. Random Forest confusion matrices comparing the species level classification accuracy of electrical diathermy-based REIMS (handheld bipolar and monopolar platform) with ALDI-MS using the 50 to 500 *m/z* range (fatty acids and low molecular weight metabolites) and 600 to 1000 *m/z* range (phospholipids). Global accuracies for each as shown in matrix notations: (**a**) 96.1%; (**b**) 89.4%; (**c**) 98.8%; (**d**) 98.8%; (**e**) 98.8%; (**f**) 99.4%.

Following optimisation of the laser operation parameters, the full repertoire of microbial isolates was analysed, with five analytical measurements taken per isolate, in line with previously reported studies^19,24^. Each replicate took approximately five seconds, allowing each isolate to be analysed in less than 45 seconds. Acquired spectral data was subjected to background subtraction, mass drift correction, total ion count normalisation, and mass binning to 0.1 Da bins, as detailed in the supporting information. Preliminary analysis using the MetaboAnalyst 3.0 platform^29^ of the 600 to 1000 *m/z* range showed that clear and significant separation was evident between the major microbial groupings, mainly bacteria and yeast, and Gram-negative and Gram-positive morphologies (Fig. 4). Although PCA modelling is a useful approach to visualise sample groupings in multivariate datasets, we have utilised the Random Forest machine learning algorithm to construct classification models validated using a leave-one-isolate-out cross validation approach. Using this method of data analysis, ALDI-MS was able to achieve a species-level classification accuracy of 99.4% (Fig. 5). This figure should be considered a measure of the conformity to MALDI-ToF based identification as no additional speciation verification, such as 16S rRNA gene sequencing, was completed. It may be therefore, that errors in MALDI-ToF identification are reflected in the classification accuracy of REIMS models as this classification is used in the creation of cross-validation models.

### Comparison of ALDI with Electrical Diathermy REIMS Modalities

In comparison to electrical diathermy REIMS, ALDI was capable of higher species-level classification accuracy than previously reported methods^19,22,23^ (Table 1 and Fig. 5). Of particular interest, ALDI-MS was shown to be capable of substantially improved species-level classification within the 50 to 500 *m/z* range, which typically contains fatty acids and low molecular weight metabolites. Although electrical diathermy REIMS employing bipolar probes was capable of reasonable accuracy within this range, the automated high-throughput monopolar REIMS platform was not. We have previously suggested that the electrical current density between these two electrical diathermy methods is substantially different. The bipolar REIMS approach is capable of an even distribution between the two probes, whilst the high-throughput monopolar platform has a high current density at the point of contact between the probe and sample, which quickly dissipates. This may result in suboptimal ionisation of a biomass and thus explain why substantially poorer, by approximately ten percentage points, classification accuracies are obtained when using the 50 to 500 *m/z* range (Fig. 5b).

**Table 1:** Precision, Sensitivity, and F1 Scores from Random Forest Species Classification.

Species	Hand-Held Bipolar		High-Throughput Monopolar		ALDI-MS	
	50 to 500	600 to 1000	50 to 500	600 to 1000	50 to 500	600 to 1000
CALB	97	100	100	100	100	100
CDIF	97	100	100	100	100	100
ECOL	94	94	79	97	97	100
HINF	100	100	83	100	100	100
KPNE	100	97	88	97	97	97
LACJ	90	100	84	97	100	100
PAER	100	100	100	100	100	100
PMIR	93	97	90	100	97	100
SAUR	100	100	97	100	97	97
SPNE	90	100	73	97	100	100
Average	96.1	98.8	89.4	98.8	98.8	99.4

The species-level classification accuracy using Random Forest for REIMS using electrical diathermy and ALDI-MS thermal disruption mechanisms for ten species is shown. For each species and thermal disruption mechanism, the classification accuracy score for mass ranges of 50 to 500 *m/z* (fatty acids and metabolites) and 600 to 1000 *m/z* (complex lipids) are shown. Full taxonomic names for each species abbreviation are given in the supporting information.

Unsupervised PCA modelling of species-level mass spectra obtained using the three REIMS approaches (Supplementary Fig. S3a–j) show that for the vast majority of microbial species analysed, ALDI-MS produced significantly different mass spectra as compared to the electrical diathermy REIMS approaches. This is likely a result of the increase in spectral intensities obtained using ALDI-MS. This is also supported by the observation that the microbial species displaying the highest degree of differences between REIMS methods are those which typically produce low biomass during normal growth, such as *Streptococcus pneumoniae*, and *Lactobacillus jensenii*.

### Cross-Validation of REIMS Modalities Classification Models

To further compare the similarity between mass spectra, each of the Random Forest classification models (Fig. 5) were tested using data from the two other REIMS modalities (Table 2). For this comparison, if a high level of classification accuracy was achieved using the processed mass spectral data from one REIMS modality against a classification model constructed using mass spectral data collected using a different modality, then the two would be considered similar. If a low level of classification was observed however, then the two REIMS modalities would be considered discordant. Through this approach, the 600 to 1000 *m/z* range showed very high levels of similarity between all three REIMS modalities; particularly between ALDI-MS and bipolar electrical diathermy, achieving an accuracy of 97%. For monopolar REIMS, an accuracy of 84% was achieved. In the lower 50 to 500 *m/z* range, reduced similarity was observed, but ALDI-MS provided the basis for the most accurate classification models. For bipolar REIMS, the laser classification model correctly classified 82% of isolates within this range. However, for monopolar REIMS, only an accuracy of 41% was achieved. This suggests that there is a high degree of similarity within the lower mass region for bipolar REIMS and ALDI-MS, but not for monopolar REIMS.

**Table 2:** Cross-Validation of REIMS Modality Classification Models.

Model → ↓Test	50 to 500 *m/z*			600 to 1000 *m/z*		
	ALDI-MS	Bipolar REIMS	Monopolar REIMS	ALDI-MS	Bipolar REIMS	Monopolar REIMS
ALDI-MS	N/A	82%	41%	N/A	97%	85%
Bipolar REIMS	74%	N/A	38%	88%	N/A	86%
Monopolar REIMS	51%	62%	N/A	97%	93%	N/A

To determine the similarity of speciation classification models created using Random Forest analysis of REIMS mass spectra acquired using the three REIMS modalities described. Here, a speciation classification model was created using training data for both mass ranges for each REIMS modality and the processed mass spectral data from the remaining two modalities used as a test set for the model. The resulting classification accuracy for each of the 12 comparisons shows high similarity between all three modalities within the complex lipid region (600 to 1000 *m/z*), but low similarity within the fatty acid and metabolite region (50 to 500 *m/z*).

### Concluding Remarks

Here we have reported on the first utilisation of ALDI mass spectrometry for species-level classification of microbial species, which shows substantially improved classification accuracy and scope for increased analytical throughput. Although we have employed a handheld surgical CO_2_ laser, the nature of the instrumentation design makes it simple to integrate into our previously reported automated and high-throughput REIMS platform^19,24^. As the utilisation of ALDI-MS employs radiative heating of the surface of a sample from a distance, it removes the necessity for sample contact and thus the requirement to change analysis probes between sampling points - potentially increasing analytical throughput of the platform. Furthermore, the use of radiative heating instead of electrical diathermy will also increase the range of sample types which can be analysed, expanding the possibility of direct-from-sample pathogen detection.

## Experimental Section

### Culturing of Microorganisms

For REIMS analysis, a total of 150 microbial isolates (15 isolates collected from distinct clinical patients from ten microbial species – nine bacterial and one yeast) were cultured according to conditions outlined in the accompanying supporting information. All isolates were originally collected from diagnostic samples received by the Imperial College NHS Healthcare Trust Diagnostic Microbiology laboratory at Charing Cross Hospital, London, after the completion of standard identification workflows. Species level identifications were confirmed using matrix assisted laser desorption ionisation time-of-flight mass spectrometry (MALDI-ToF) as previously described^19,24^.

### ALDI-MS Analysis using Xevo G2-XS qTOF

Isolates were analysed using a helium gas-cooled CO_2_ surgical laser (FELS-25A, OmniGuide, USA), which utilises a fibre optic beam guide^30^, with a wavelength of 10.6 µm, and a gas pressure of 30 psi. The resultant analyte-containing vapour was aspirated to a Xevo G2- XS QToF mass spectrometer (Waters Corporation, UK) operated in negative ion detection mode and under parameters as previously described^19,24^ (Fig. 1) and which are also detailed in the supporting information.

### Use of Previously Published Electrical Diathermy REIMS Data

Here, ALDI-MS data is compared to previously published electrical diathermy REIMS data^19^. This data was collected as previously described using the same mass spectrometry instrument and operating parameters as detailed for the ALDI-MS analysis shown here. This dataset consisted of 25 microbial species, ten of which were analysed using ALDI-MS in this study. The raw data for the subset of ten species used in this ALDI-MS study were extracted and processed using the parameters detailed below.

### Data and Statistical Analysis

To correct for mass drift and remove background mass spectral signals, all.RAW data files were processed using MassLynx software (V4.1, Waters) through the Accurate Mass Measure tool employing the Automatic Peak Detection option with all options chosen, with lock mass correction against a mass of 554.2615 with a mass windows of +/− 0.500, and averaged over a total of ten scans. Resulting processed mass spectra were used in subsequent intensity analysis calculations, identification of most intense spectral peaks, and *m/z* values for accurate mass identification of metabolites. For intensity analysis, mean and standard deviations were calculated from individual intensity values taken for five identified analytical repeats identified through total ion count peaks within the acquisition file.

Additionally, raw mass spectral data files were processed using the Offline Model Building (OMB) software (Version 1.1.29.0, Waters) to perform background subtraction, mass drift correction against leucine encephalin lock mass compound (negative mode *m/z* = 554.2615), and mass binning to 0.1 Da within chosen restricted mass ranges. For statistical analysis, including principal component analysis (PCA), the MetaboAnalyst 3.0^29^ platform was used. After processing of mass spectra, a data matrix was exported from the OMB software and uploaded to the MetaboAnalyst 3.0 online analysis pipeline where it underwent additional processing for log transformation and Pareto scaling (mean-centred and divided by the square root of standard deviation of each variable). For PCA analysis, the first and second principal components were used in two dimensional plots, with 95% confidence regions displayed using colour shading.

For Random Forest analysis, a mass spectral matrix after data pre-processing in the Offline Model Builder Software was imported into the machine learning scikit-learn package^31^, via a bespoke graphical user interface. This interface utilised Cytoscape.js^32^ and plotly.js packages for data visualisation. This package was used for both sample classification and identification of important mass spectral bins used in sample classification models. A total of 400 decision making trees were used in the construction of each classification model, and the Random Forest algorithm expanded all trees until the ‘leaves’ were considered pure. Leave-one-isolate-out cross-validation was completed to determine the classification accuracy of each Random Forest model.

### Safety Considerations

Throughout the work detailed here, all microbial isolates were treated as potential Hazard Group 2 organisms and thus, were handled within containment level two facilities. All REIMS analysis was completed within a class two biological safety cabinet. All chemicals and solvents used in this work were handled according to their material safety data sheet provided by the relevant manufacturer. The CO_2_ laser used in this work is classified as Class IVb and thus safety eyewear, matched to the wavelength of the laser, were used whilst conducting the experimental work, in additional to all other safety precautions recommended by the manufacturer.

## Supplementary information

Supplementary Information
