# Supplementary material for: Utilisation of Ambient Laser Desorption Ionisation Mass Spectrometry (ALDI-MS) Improves Lipid-Based Microbial Species Level Identification
Source: Sci Rep. 2019 Feb 28;9:3006. doi: 10.1038/s41598-019-39815-w (PMC6395639; doi:10.1038/s41598-019-39815-w)

Supplementary Information

Utilisation of Ambient Laser Desorption Ionisation Mass Spectrometry (ALDI-MS) Improves Lipid-Based Microbial Species Level Identification

Simon J.S. Cameron (1)†, Zsolt Bodai (1)†, Burak Temelkuran (2), Alvaro Perdones-Montero (1), Frances Bolt (1), Adam Burke (1), Kate Alexander-Hardiman (1), Michel Salzet (4), Isabelle Fournier (4), Monica Rebec (3), Zoltán Takáts (1)*.

† Contributed equally

* Communicating author: Prof. Zoltan Takáts. Email: z.takats@imperial.ac.uk

(1) Division of Computational and Systems Medicine, Department of Surgery and Cancer, Imperial College London, South Kensington Campus, London, UK.

(2) The Hamlyn Centre, Department of Computing, Faculty of Engineering, Imperial College London, South Kensington Campus, London, UK.

(3) Department of Microbiology, Imperial College Healthcare NHS Trust, Charing Cross Hospital, London, UK.

(4) Laboratoire Proteomique, Reponse Inflammatoire et Spectrometrie de Mass (PRISM), Universite de Lille, Lille, France.

Contents

1 Table S1 - Microbial Isolates and Culture Conditions

2 Table S2 - Xevo G2-XS QToF Instrument Operation Parameters

3 Figure S1 - Spectral Differences between Electrical Diathermy and LASER-Ablation REIMS

[1] Table S1 – Microbial Isolates and Culture Conditions

Information on the ten microbial species used in this work, including the taxonomic classifications of each, and the culture conditions used for each of the fifteen isolates of each species. CBA = Colombia Blood Agar.

**Taxonomic Classification**				**Culture Conditions**			
**Genus**	**Species**	**Domain**	**Gram Morphology**	**Media**	**Temperature (°C)**	**Atmosphere**	**Duration (hrs)**
Candida	*albicans*	Fungi	N/A	CBA	30	Aerobic	48
Clostridium	*difficile*	Bacteria	Positive	CBA	37	Anaerobic	48
Escherichia	*coli*	Bacteria	Negative	CBA	37	Aerobic	24
Haemophilus	*influenza*	Bacteria	Negative	Chocolate	37	CO_2	48
Klebsiella	*pneumoniae*	Bacteria	Negative	CBA	37	Aerobic	24
Lactobacillus	*jensenii*	Bacteria	Positive	CBA	37	$C0_2$	48
Pseudomonas	*aeruginosa*	Bacteria	Negative	CBA	37	Aerobic	24
Proteus	*mirabilis*	Bacteria	Negative	CBA	37	Aerobic	24
Staphylococcus	*aureus*	Bacteria	Positive	CBA	37	Aerobic	24
Streptococcus	*pneumonia*	Bacteria	Positive	CBA	37	$C0_2$	48

[2] Table S2 – Xevo G2-XS QToF Instrument Operation Parameters

Instrumentation operation parameters of the Xevo G2-XS QToF instrument used during sample analysis are given.

Parameter	Setting
Scan Time	1000 ms
Scan Mode	Sensitive
Mass Analyser	Time of Flight
Ion Detection Mode	Negative
Mass Range (*m/z*)	50 to 2500
Sampling Cone	80 V
Source Offset	50 V

[3] Figure S1a – Spectral Differences between Electrical Diathermy and ALDI-MS within *Candida albicans* Isolates

PCA plot of spectral data (50 to 2500 *m/z*) comparing the three different REIMS modalities and mean spectral comparisons between ALDI-MS (Laser REIMS) and bipolar REIMS and between ALDI-MS and automated high-throughput monopolar REIMS.

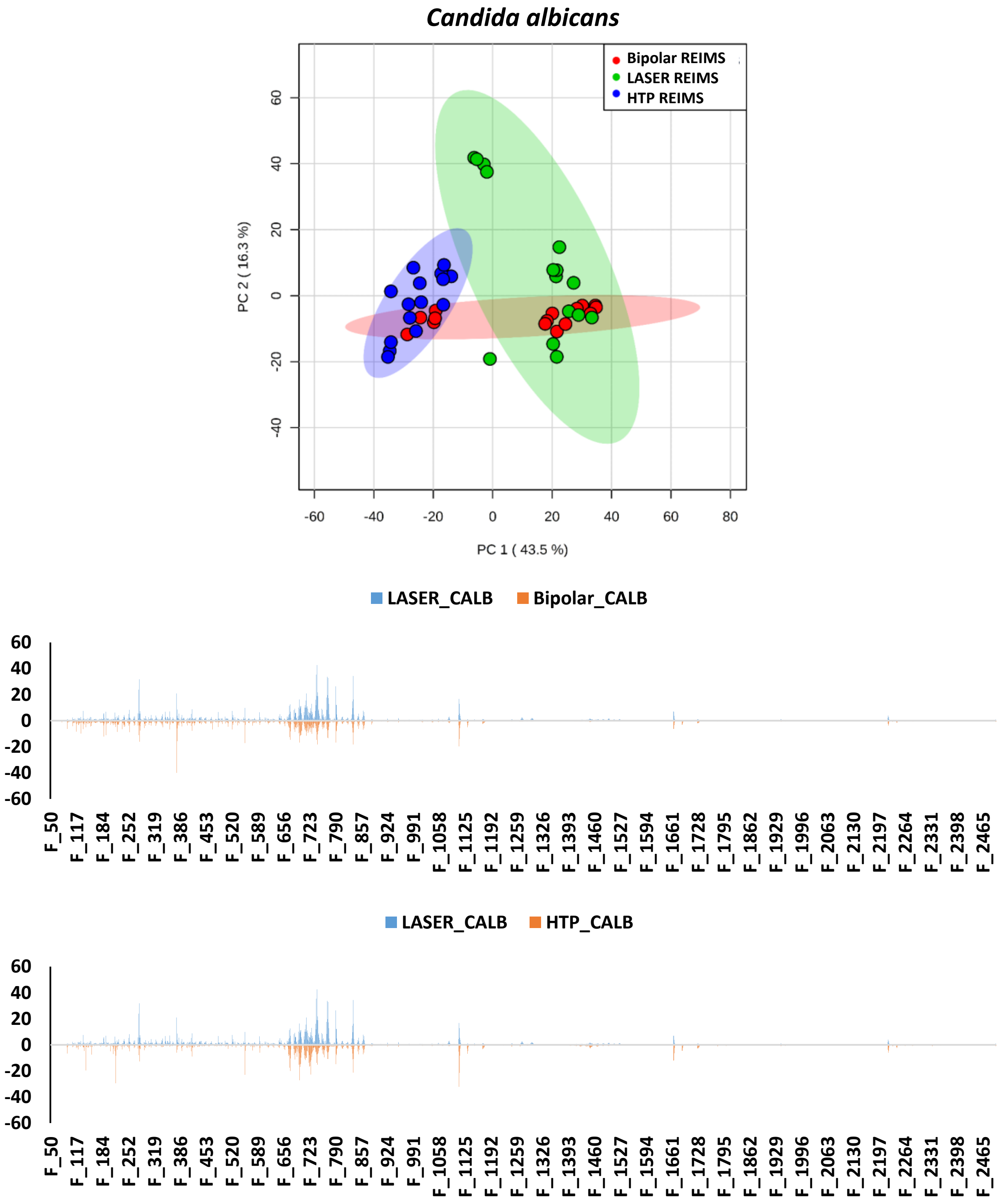

[3] Figure S1b – Spectral Differences between Electrical Diathermy and ALDI-MS within *Clostridium difficile* Isolates

PCA plot of spectral data (50 to 2500 m/z) comparing the three different REIMS modalities and mean spectral comparisons between ALDI-MS (Laser REIMS) and bipolar REIMS and between ALDI-MS and automated high-throughput monopolar REIMS.

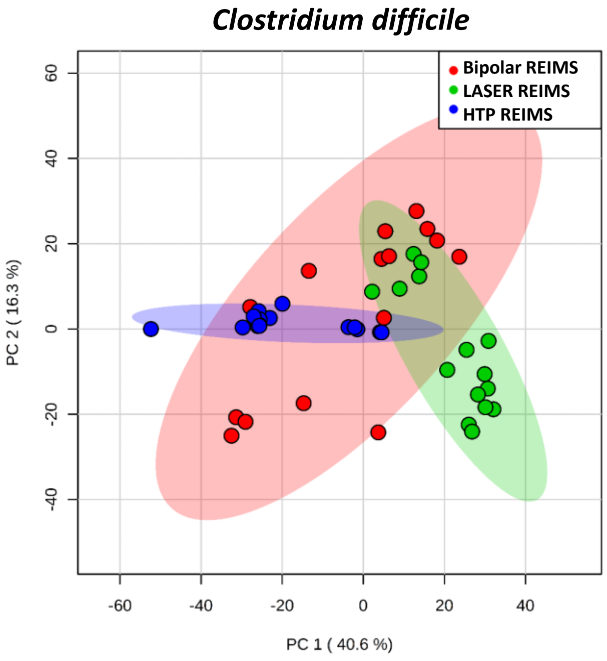

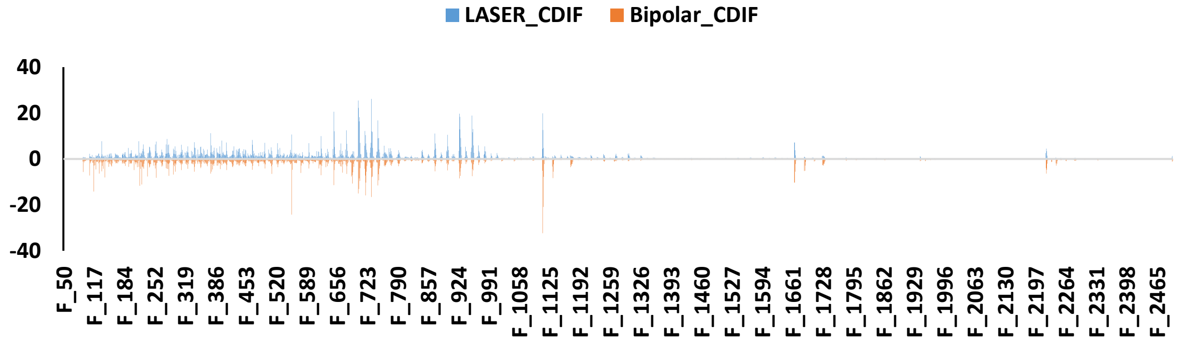

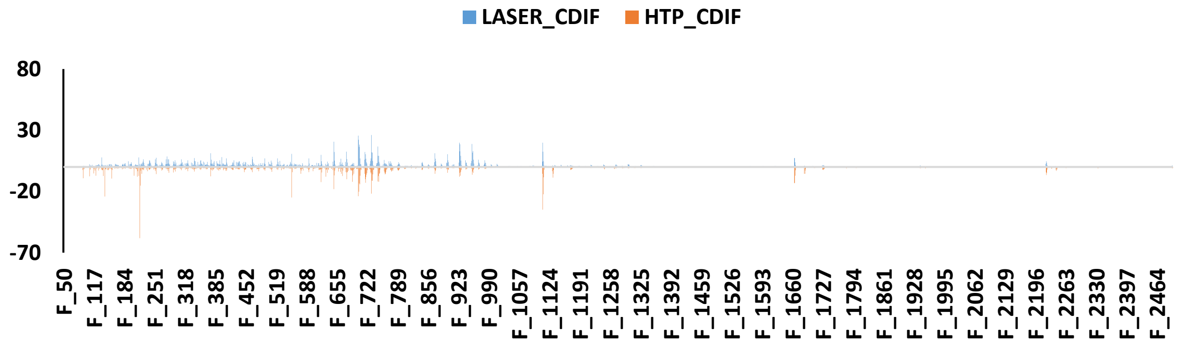

[3] Figure S1c – Spectral Differences between Electrical Diathermy and ALDI-MS within *Escherichia coli* Isolates

PCA plot of spectral data (50 to 2500 *m/z*) comparing the three different REIMS modalities and mean spectral comparisons between ALDI-MS (Laser REIMS) and bipolar REIMS and between ALDI-MS and automated high-throughput monopolar REIMS.

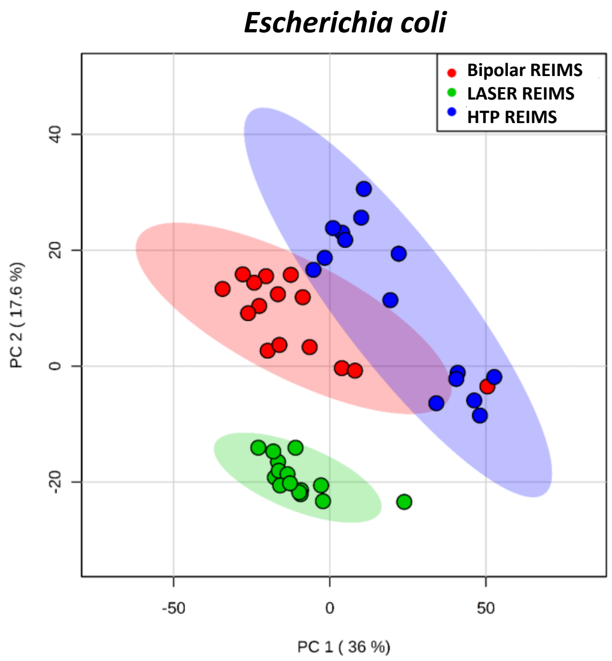

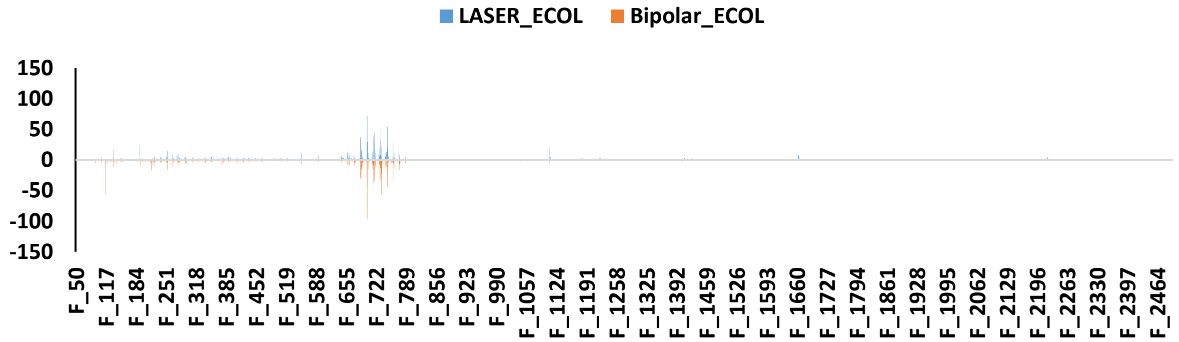

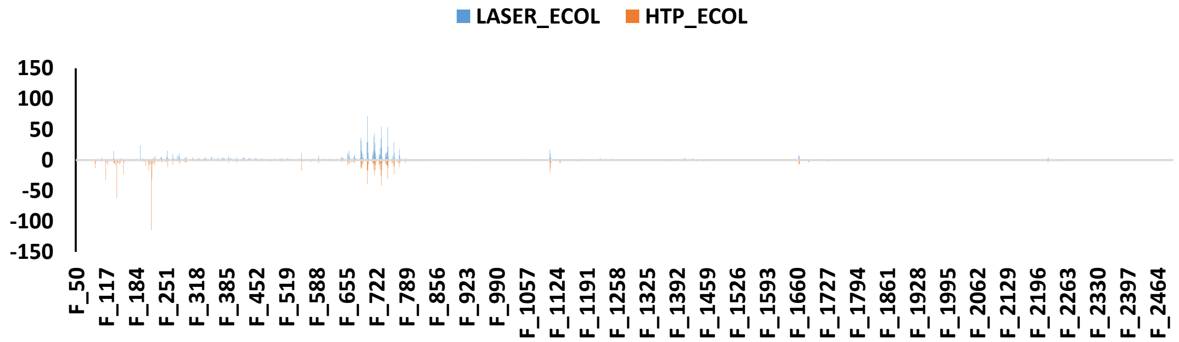

[3] Figure S1d – Spectral Differences between Electrical Diathermy and ALDI-MS within *Haemophilus influenzae* Isolates

PCA plot of spectral data (50 to 2500 *m/z*) comparing the three different REIMS modalities and mean spectral comparisons between ALDI-MS (Laser REIMS) and bipolar REIMS and between ALDI-MS and automated high-throughput monopolar REIMS.

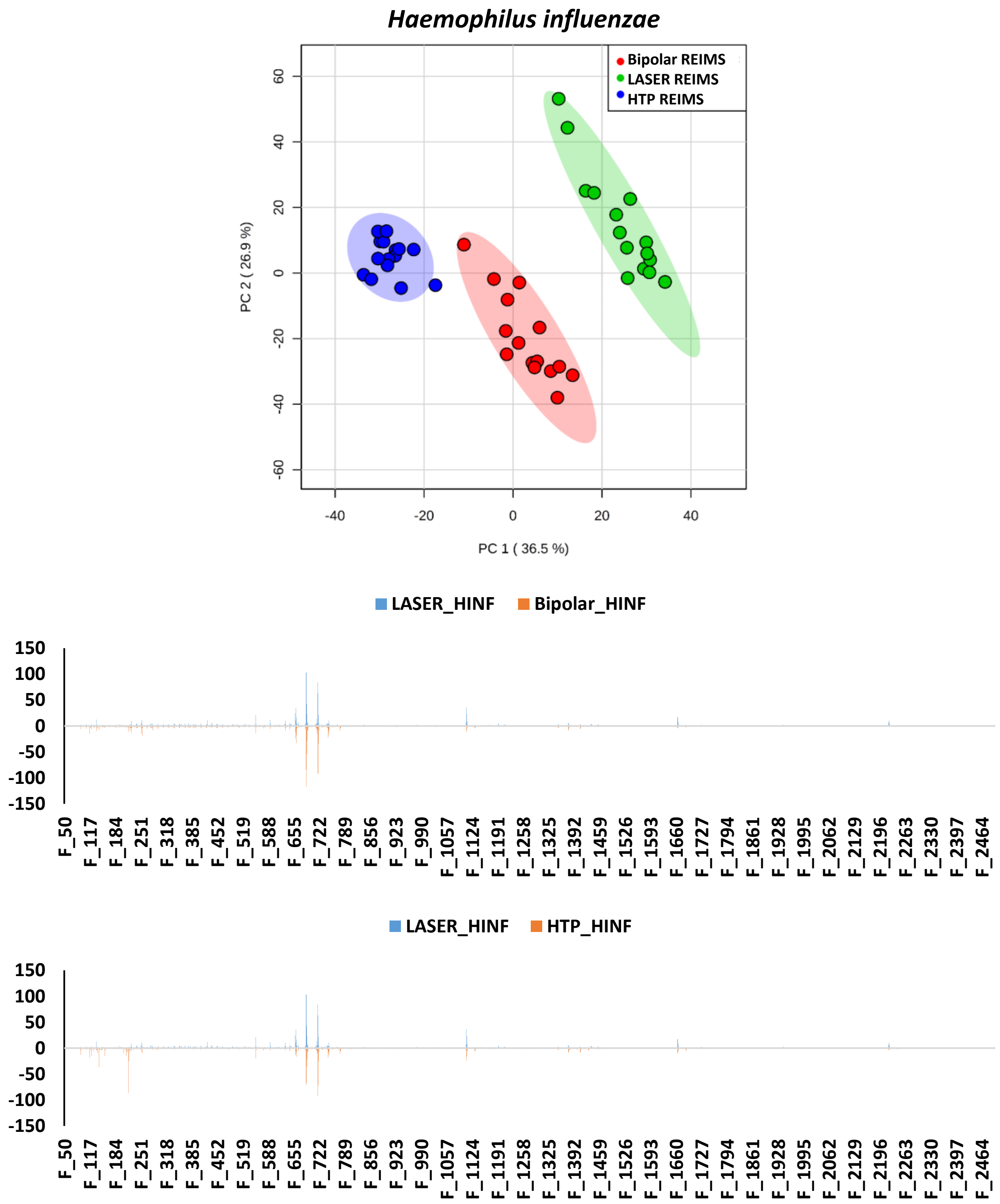

[3] Figure S1e – Spectral Differences between Electrical Diathermy and ALDI-MS within *Klebsiella pneumoniae* Isolates

PCA plot of spectral data (50 to 2500 *m/z*) comparing the three different REIMS modalities and mean spectral comparisons between ALDI-MS (Laser REIMS) and bipolar REIMS and between ALDI-MS and automated high-throughput monopolar REIMS.

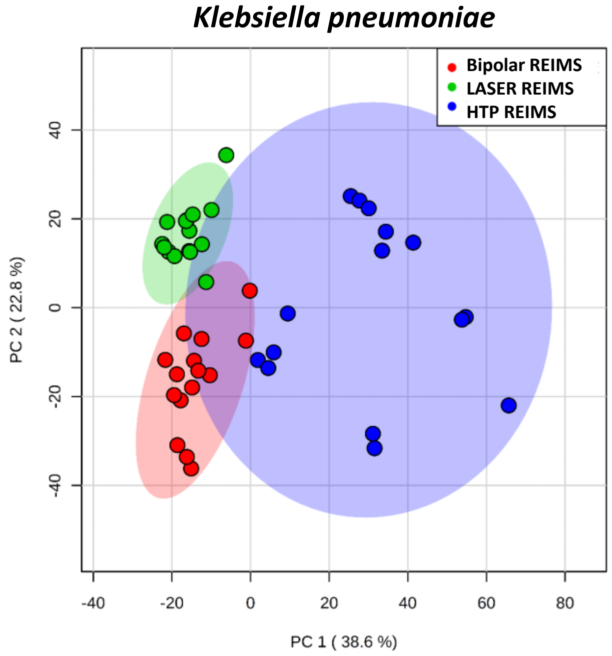

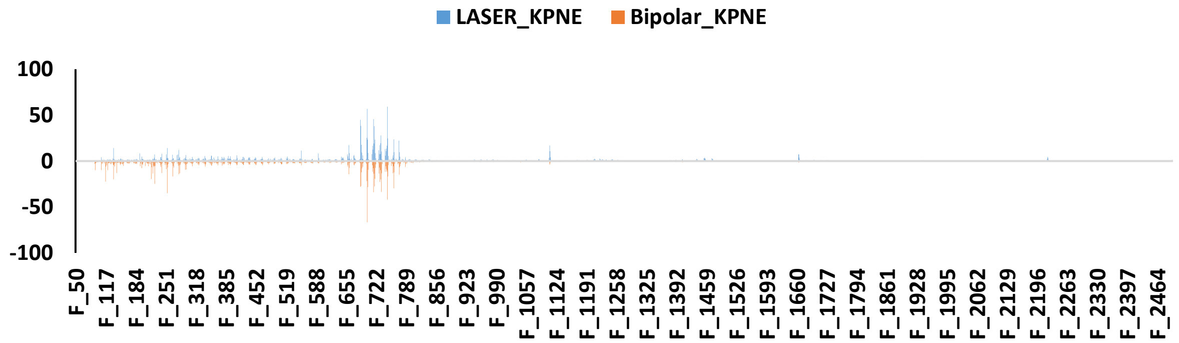

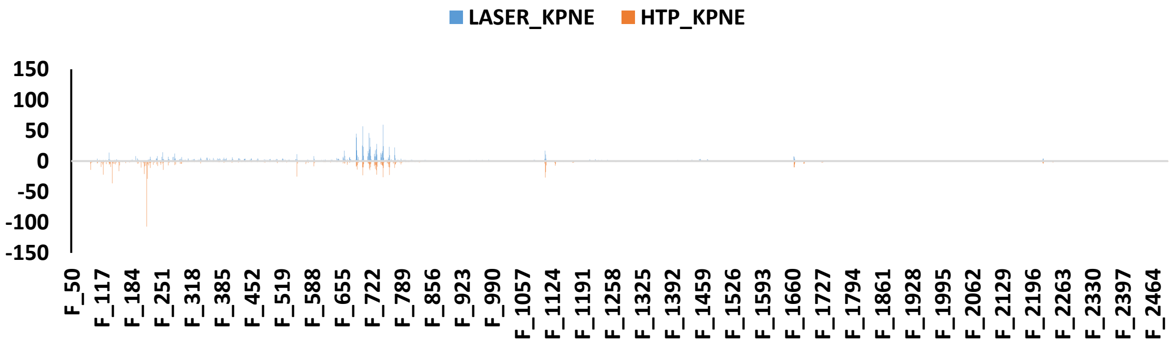

[3] Figure S1f – Spectral Differences between Electrical Diathermy and ALDI-MS within *Lactobacillus jensenii* Isolates

PCA plot of spectral data (50 to 2500 *m/z*) comparing the three different REIMS modalities and mean spectral comparisons between ALDI-MS (Laser REIMS) and bipolar REIMS and between ALDI-MS and automated high-throughput monopolar REIMS.

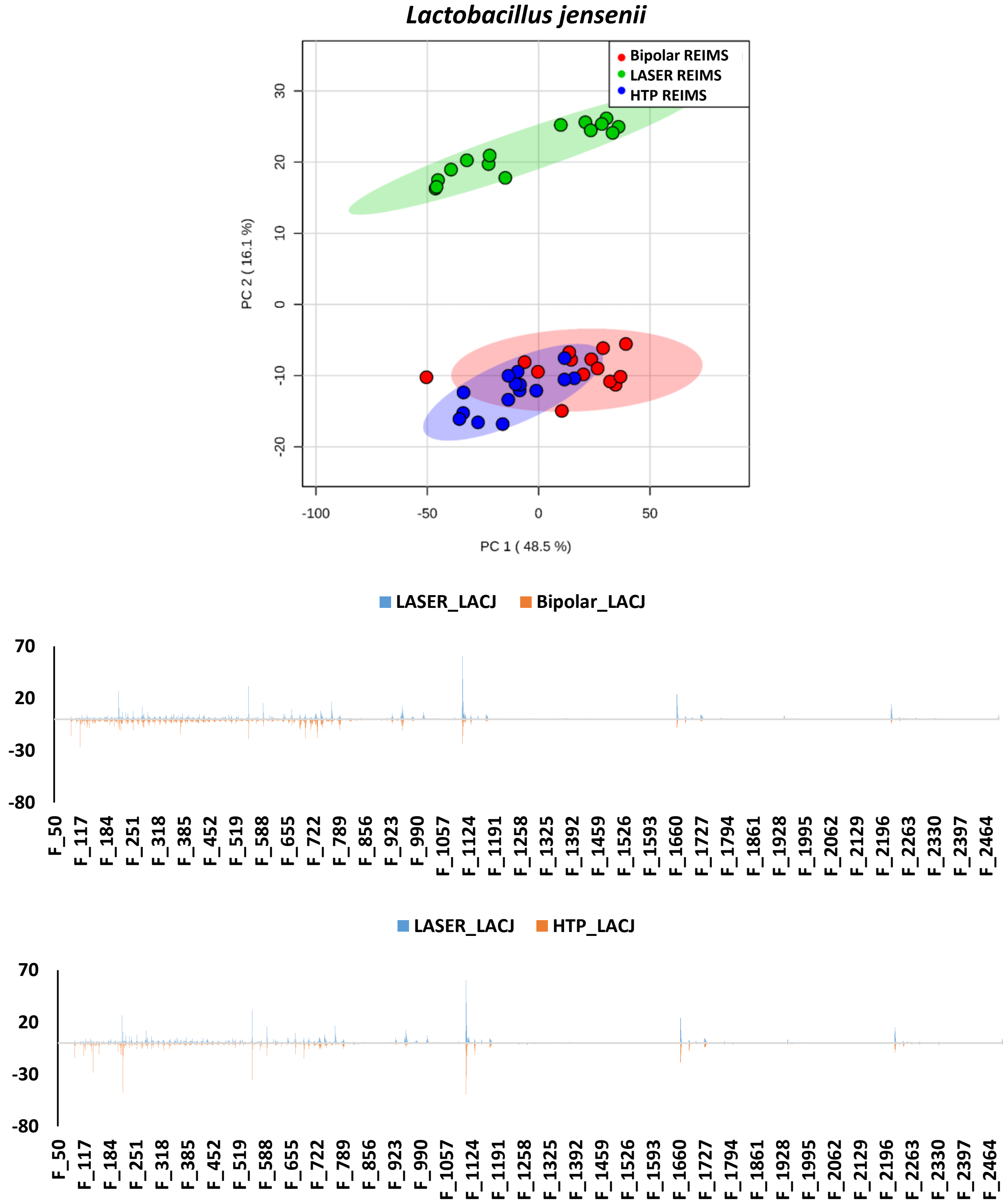

[3] Figure S1g – Spectral Differences between Electrical Diathermy and ALDI-MS within *Pseudomonas aeruginosa* Isolates

PCA plot of spectral data (50 to 2500 *m/z*) comparing the three different REIMS modalities and mean spectral comparisons between ALDI-MS (Laser REIMS) and bipolar REIMS and between ALDI-MS and automated high-throughput monopolar REIMS.

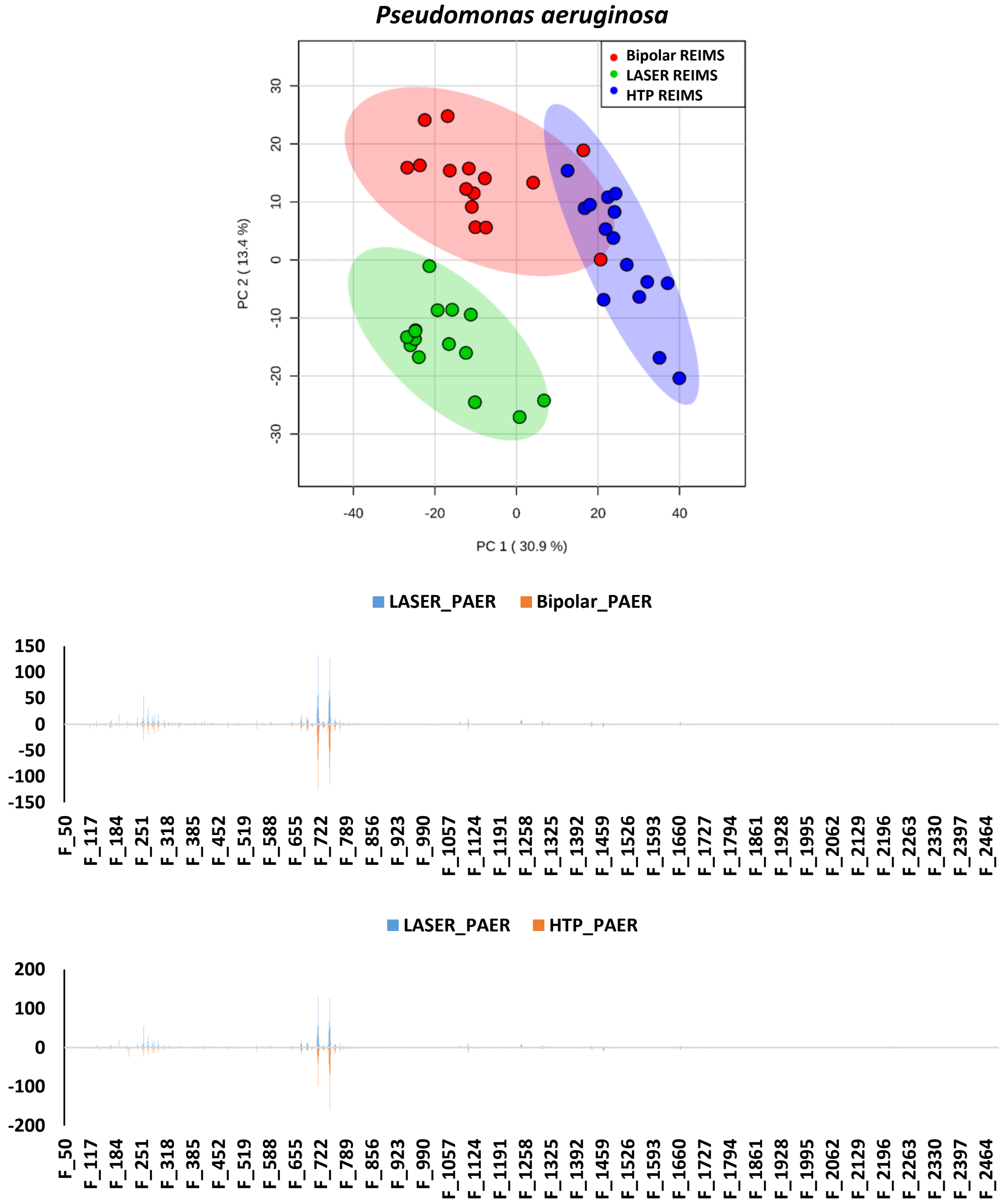

[3] Figure S1h – Spectral Differences between Electrical Diathermy and ALDI-MS within *Proteus mirabilis* Isolates

PCA plot of spectral data (50 to 2500 *m/z*) comparing the three different REIMS modalities and mean spectral comparisons between ALDI-MS (Laser REIMS) and bipolar REIMS and between ALDI-MS and automated high-throughput monopolar REIMS.

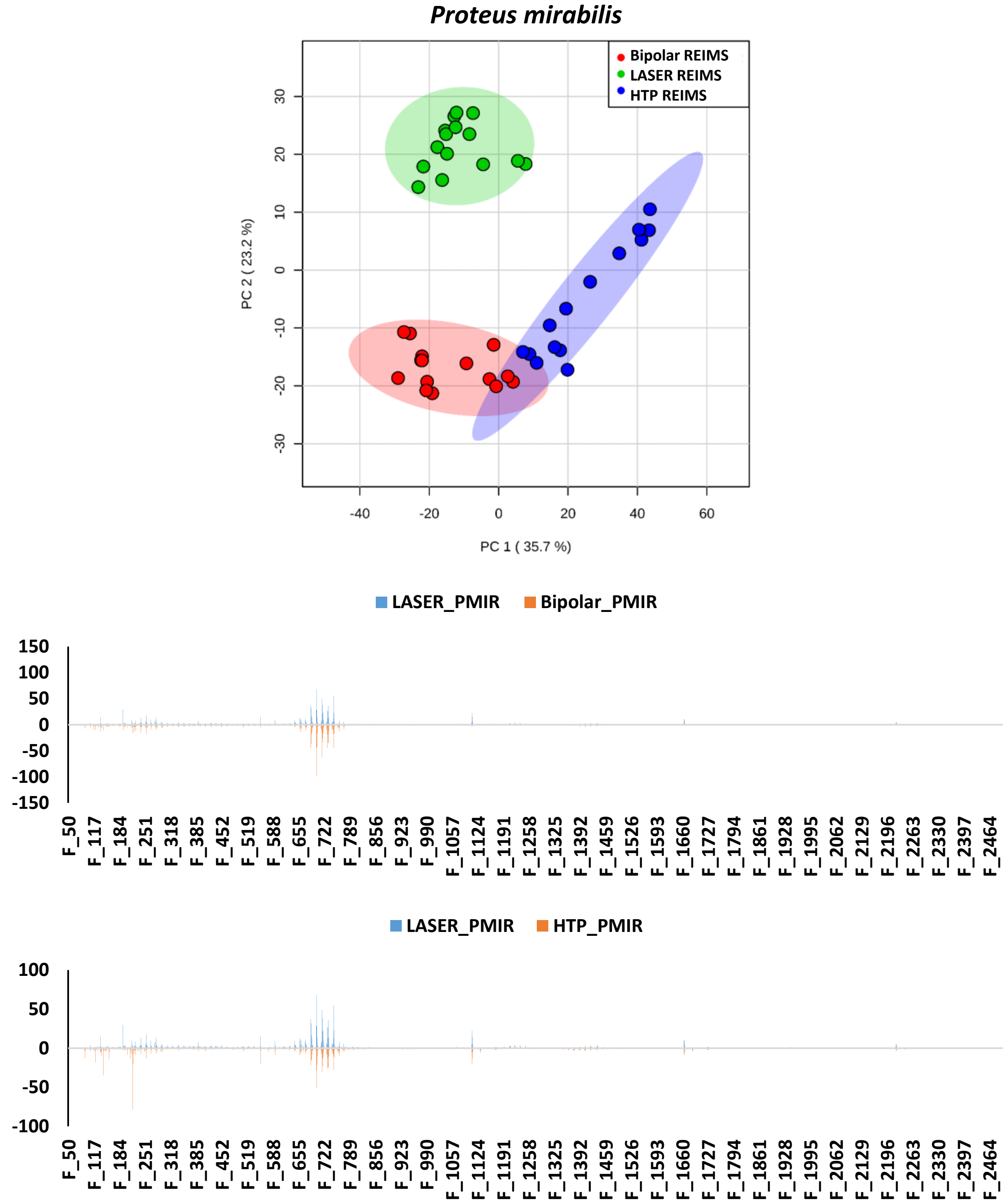

[3] Figure S1i – Spectral Differences between Electrical Diathermy and ALDI-MS within *Staphylococcus aureus* Isolates

PCA plot of spectral data (50 to 2500 *m/z*) comparing the three different REIMS modalities and mean spectral comparisons between ALDI-MS (Laser REIMS) and bipolar REIMS and between ALDI-MS and automated high-throughput monopolar REIMS.

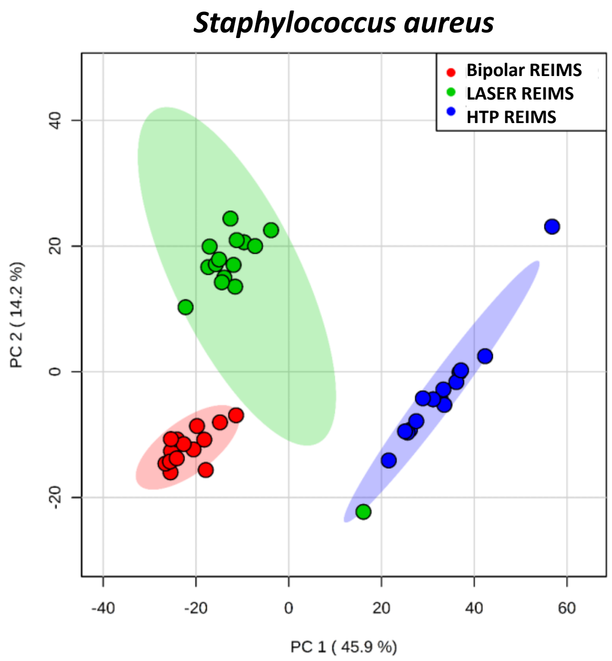

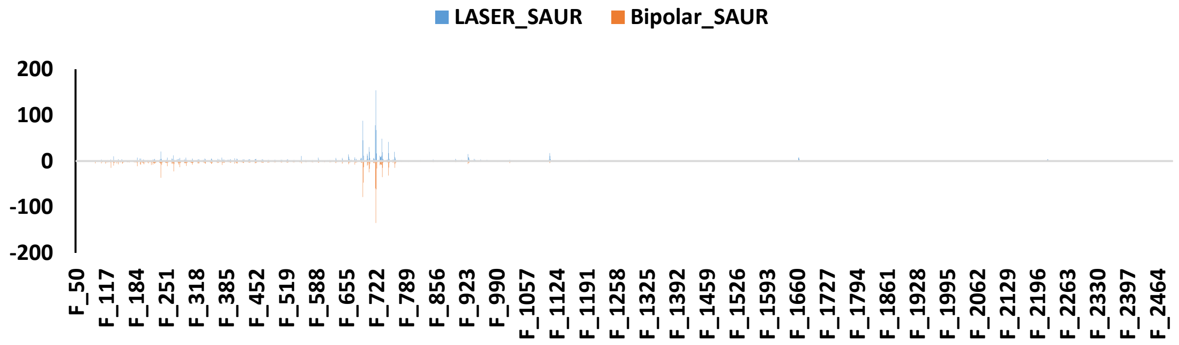

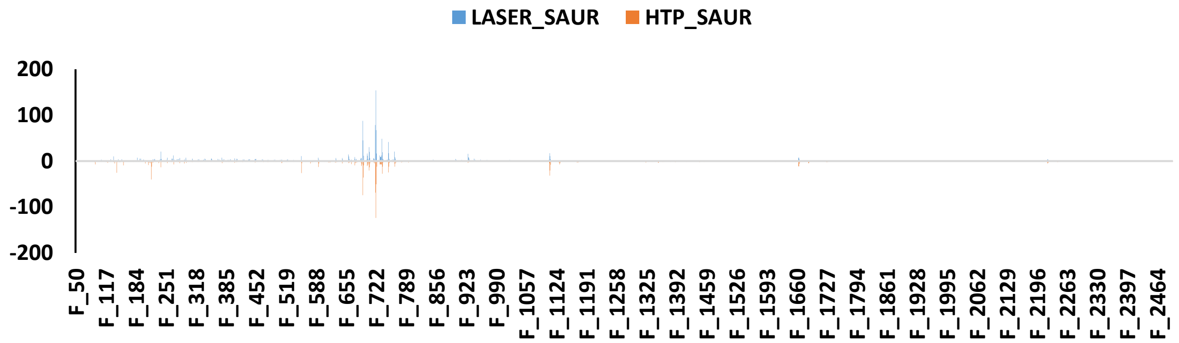

[3] Figure S1j – Spectral Differences between Electrical Diathermy and ALDI-MS within *Streptococcus pneumoniae* Isolates

PCA plot of spectral data (50 to 2500 *m/z*) comparing the three different REIMS modalities and mean spectral comparisons between ALDI-MS (Laser REIMS) and bipolar REIMS and between ALDI-MS and automated high-throughput monopolar REIMS.

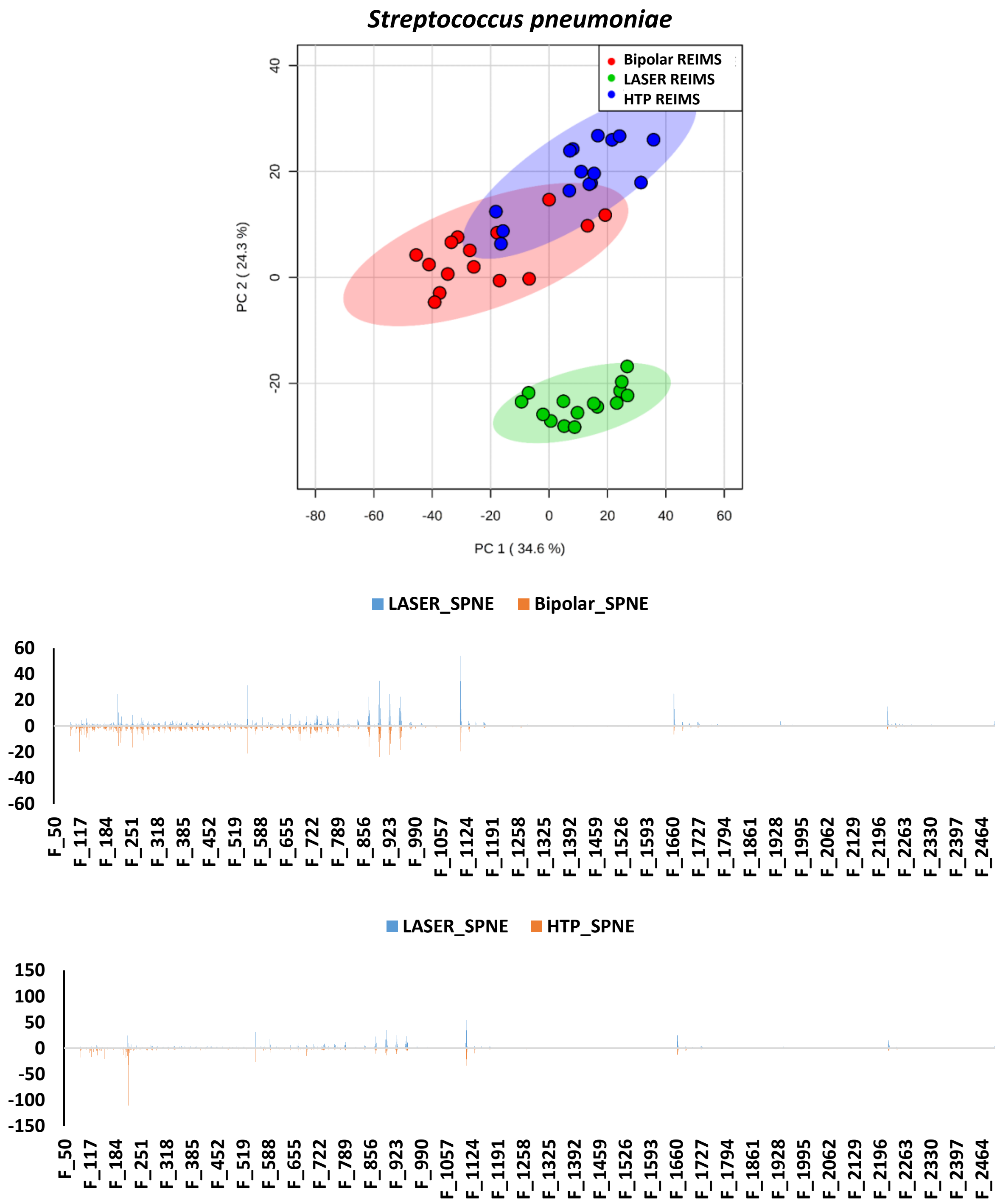

Supplement: Supplementary file 1 — Supplementary Information [file 41598_2019_39815_MOESM1_ESM.pdf]
